# The influence of chlorogenic acid disinfection on the oral microbiota of alginate impressions

**DOI:** 10.1080/20002297.2026.2727218

**Published:** 2026-09-03

**Authors:** Tiehan Cui, Meichun Hu, Shuling Jiang, Fuqing Chen, Qiaoqiao Hu, Liping Yang, Na Li

**Affiliations:** a Department of Stomatology, The First Affiliated Hospital of Nanchang University, Nanchang, China; b Loudi Vocational and Technical College, Loudi, China

**Keywords:** Chlorogenic acid, alginate impression, oral microbiota, 16S rRNA gene sequencing, disinfection

## Abstract

**Background:**

Cross-infection may occur if alginate impressions are not disinfected. Available chlorine (AC), though common, poses safety risks. Chlorogenic acid (CA), a natural compound, shows effective disinfection and, as we previously verified, inhibits pathogens without damaging impressions.

**Objective:**

This study investigates the influence of CA and AC on the oral microbial community of alginate impressions from a community-ecology perspective.

**Methods:**

The maxillary impressions of 60 participants were randomly assigned to four groups (*n* = 15). The NS-AC group received a normal saline (NS) rinse and a 2,000 mg/L AC spray. The NS-CA group got an NS rinse and a CA (10 mg/mL mix + 60 mg/mL spray). The CA-AC group obtained a 10 mg/mL CA rinse and a 2,000 mg/L AC spray. The CA-CA group acquired a CA (10 mg/mL rinse & mix + 60 mg/mL spray). An analysis of 16S rRNA sequencing was conducted on the samples that were collected.

**Results:**

The NS-CA group exhibited substantially higher Chao1, Shannon and Simpson indices than the NS-AC group (*p* < 0.05), and the PCoA revealed a centralized clustering pattern. As the primary biomarkers in the NS-CA, *Neisseria*, *Porphyromonas* and *Prevotella* were identified through LEfSe analysis and a random forest model (LDA > 4, AUC = 0.911). A synchronous decrease in the NS-CA was predicted by FAPROTAX functional prediction in relation to *aromatic_compound_degradation* and *human_pathogens_all*. The NS-CA and CA-CA did not exhibit any significant differences in alpha diversity or FAPROTAX (*p* > 0.05), and the PCoA showed that the two groups were closely clustered.

**Conclusion:**

The stabilization and maturation of oral microbiota on the surface of alginate impressions are facilitated by CA disinfection. Of all the groups, the NS-CA group demonstrates specific cost-effectiveness advantages and has the potential to serve as an alternative to conventional AC disinfection.

## Key messages

- CA disinfection causes the alginate impression to be dominated by oral commensal bacteria, presenting characteristics of increased diversity and balanced structure.
- AC disinfection causes the alginate impression to be dominated by opportunistic pathogens, presenting characteristics of reduced diversity and disordered structure.
- The NS-CA group has certain advantages in terms of cost-effectiveness and may become one of the alternative options for traditional AC disinfection.

## Introduction

Alginates are among the most frequently employed impression materials in dentistry due to their favorable properties, which include affordability, precision, non-reversibility and elasticity [1,2]. They are frequently utilized in procedures such as orthodontic treatment and the fabrication of removable dentures. Nevertheless, alginate impressions are more susceptible to the retention of a significant number of bacteria, viruses and fungi from saliva and blood following their removal from the buccal cavity due to their porous, rough and hydrophilic physical structure [3,4]. The potential cross-infection risk to dental practitioners, auxiliaries and laboratory technicians is posed by the potential for these impressions to act as a vector for microbial transmission [5,6]. Consequently, it is imperative to ensure the safety of dental personnel and prevent the spread of infection by effectively disinfecting alginate impressions.

A globally unified ‘gold standard’ for alginate impression disinfection has not yet been established, despite its significance. The primary practical methods currently in use involve the incorporation of chemical disinfection or antibacterial nanomaterials [6,7]. Nevertheless, research has demonstrated that chemical disinfection can result in skin allergies or respiratory mucosal irritation in operators, while nanomaterials not only accrue high production costs but also produce potentially toxic by-products [8–12]. Chemical disinfection, despite its extensive use, is not suitable for long-term application due to these drawbacks and the high cost and toxicity of nanomaterials impede their widespread adoption [13,14]. Consequently, the ongoing challenge in dental practice is the pursuit of a more natural, efficient and cost-effective impression disinfectant.

In recent years, chlorogenic acid (CA), a natural compound extracted from honeysuckle and coffee beans, has been widely studied for its antioxidant, anti-inflammatory, antibacterial and antiviral properties [15–17]. CA has exhibited substantial potential in a variety of oral health applications, such as the inhibition of oral bacterial growth and colonization [18], the promotion of periodontal tissue repair and regeneration [19] and the suppression of bacterial growth in root canals [20]. Nevertheless, its utilization in alginate impressions has garnered relatively little attention.

Until now, our research has been the sole investigation into the impact of CA on the dimensional accuracy, physical properties and antibacterial efficacy of alginate impressions [21]. The growth of *Escherichia coli*, *Staphylococcus aureus*, *Candida albicans* and *Streptococcus pneumoniae* on the alginate impression was completely inhibited by a combined protocol featuring 10 mg/mL CA mixing, followed by a 30-minute application of 60 mg/mL CA spray, as compared to the conventional clinical disinfection method with 2,000 mg/L available chlorine (AC) spray, as demonstrated in our previous studies. Additionally, the influences on the physical properties of the impression and the precision of the poured gypsum models were within clinically acceptable limits. Furthermore, Singer et al. [22] and Masih et al. [23] have conducted research that has shown that mixing and spraying are effective methods for disinfecting alginate impressions and can reduce impression deformation to the utmost extent. These results serve as a foundation for the intervention strategies implemented in the current investigation.

Despite the fact that CA has been demonstrated to effectively impede bacterial growth on the surface of alginate impressions, its widespread use as a disinfectant has not yet been achieved and requires additional scientific evidence. Interestingly, we discovered that the composition of the microbial community of the alginate impression after disinfection was not investigated by CA disinfection, chemical disinfection, or nano-disinfection. However, the clinical value of a disinfectant may be related to the composition of the microbial community, which could influence whether the impression is at risk of infection following disinfection. The dwelling space for other foreign pathogens will be reduced if the impression is disinfected and leaves a balanced community dominated by symbiotic bacteria. In contrast, a community that is primarily populated by drug-resistant microbes will be at a greater risk of subsequent transmission.

Therefore, in order to address this gap and offer more objective, environmentally friendly and effective disinfection options for alginate impressions, the current study expands upon our previous research by employing 16S rRNA gene sequencing to further investigate the impact of CA disinfection on the oral microbial community of the impression. The objective is to validate the potential of CA disinfection as a novel disinfectant.

## Materials and methods

### Study participants

Between May 2023 and September 2023, a total of 60 dental students from the Department of Stomatology at The First Affiliated Hospital of Nanchang University were enrolled as study participants. Before inclusion, each participant executed a written informed consent form. This investigation was conducted in compliance with the Declaration of Helsinki and was authorized by the Medical Ethics Committee of the First Affiliated Hospital of Nanchang University (No. ITT [2023] Clinical Ethics Review No. 158).

The following were the inclusion criteria for study participants: (1) the gingiva is pink and healthy in texture; the full-mouth probing depths are less than 3 mm with no bleeding on probing; and there is no evidence of dental caries, periodontal attachment loss, or other oral mucosal diseases. The area covered by plaque and soft debris is less than one-third of the tooth surfaces. (2) No periodontal treatment during the past six months, and no use of systemic antibiotics, mouthwash, or antibacterial agents. (3) The oral cavity contains a sufficient number of natural teeth that are in good condition to allow for the acquisition of a qualified impression. A single examiner evaluated and documented all clinical findings to guarantee uniformity in the inclusion criteria.

The following were the exclusion criteria: (1) inability to comprehend or participate in the research procedures, including those with inadequate mouth opening to facilitate impression taking; (2) currently undergoing orthodontic treatment; (3) smoking; (4) active infectious diseases, immunodeficiency disorders, or uncontrolled systemic diseases; and (5) known allergy to honeysuckle extract, coffee beans, CA, or any other materials utilized in this investigation.

### Grouping

The maxillary impressions of the 60 enrolled participants were randomly assigned to four groups (*n* = 15): NS-AC, NS-CA, CA-AC and CA-CA in this study. The intervention protocols for each cohort were as follows:

(1) NS-AC group: normal saline (NS) rinse + 2,000  mg/L AC spray.
(2) NS-CA group: NS rinse and CA (10 mg/mL mix + 60 mg/mL spray).
(3) CA-AC group: 10 mg/mL CA rinse + 2,000 mg/L AC spray.
(4) CA-CA group: CA (10 mg/mL rinse & mix + 60 mg/mL spray).

### Sample collection

Before maxillary impression acquisition, participants in each of the four groups rinsed with their designated solution-either 25 mL of NS or 10 mg/mL CA for one minute. A single impression was then obtained from each participant.

Immediately after removal, all surfaces of each impression were evenly sprayed with the corresponding disinfectant. A total of 10 sprays were applied, with a 2-second interval between each spray to guarantee that the entire impression surface was thoroughly moistened without any visible dripping. The spray volume applied per impression was approximately 40 mL.

After spraying, the impressions were allowed to remain at room temperature for 30 min. Subsequently, they were rinsed with a sterile neutralizing agent for 30 seconds to terminate the reaction. The entire surface of each impression was further wiped with sterile cotton swabs that had been moistened with sterile distilled water. The swabs were placed into sterilized sampling tubes that contained 5 mL of sterile distilled water, without any hand-contact portions. The samples were transferred to the laboratory within 2 hours and stored at −80 °C until further analysis, after being shaken for 2~3 min.

The following were the inclusion criteria for impressions: (1) completion: the impression was entirely extracted from the buccal cavity without any significant deformation, tearing, or breakage. (2) Clarity: the anatomical structures that were crucial to the study, such as the gingival margins and teeth, were easily identifiable. (3) Timeliness: the impression was processed according to the established pre-treatment protocol within the designated time frame after removal.

The impressions were excluded based on the following criteria: (1) operational errors: the impression failed to fully set during removal or detached from the tray; (2) contamination: contact with non-oral surfaces during removal; (3) substandard quality: the presence of substantial cavities or bubbles on the impression surface.

### 16S rRNA gene sequencing

Microbial DNA was extracted from the samples in accordance with the manufacturer's instructions using the HiPure Soil DNA Mini Kit (D3142-03; Meiji Biotechnology Co., Ltd., Guangzhou, China). DNA concentration and purity were assessed using a NanoDrop One spectrophotometer (NanoDrop Technologies, Wilmington, DE, USA) and agarose gel electrophoresis. The V3-V4 hypervariable region of the 16S rRNA gene was amplified by PCR using primers 338F and 806 R, which were modified with sample-specific barcodes. High-fidelity DNA polymerase was incorporated into the PCR reaction mixture. The Qubit 3.0 Fluorometer (Life Technologies, Carlsbad, CA, USA) was used to quantify the amplicons that were purified using magnetic beads. Purified amplicons from each sample were pooled in equimolar amounts and sequenced on a PromethION sequencer (Oxford Nanopore Technologies, Oxford, UK) by Wuhan Bena Technology Co., Ltd.

### Analysis of oral microbial information

Initially, the integrity of raw sequencing data was evaluated using FastQC (v0.11.9). Porechop (v0.2.3) was employed to eliminate the barcode and adapter sequences. Sequences that were shorter than 200 bp or had an unacceptably low average quality were discarded using Trimmomatic (v0.39), with low-quality bases (Q < 10) at both extremities of the reads being trimmed. The FASTQ sequences that were processed were subsequently imported into the QIIME 2 pipeline for microbial community analysis. Ultimately, a table of ASVs and their abundance across samples was generated by employing the DADA2 module within QIIME 2 for quality control, denoising, merging of paired-end reads and chimera removal. The representative ASV sequences were taxonomically assigned using a naive Bayes classifier that was trained on the Silva database (v138).

The initial assessment of species composition was conducted at the ASV level. The relative abundance histograms at the phylum and genus levels were employed to compare community structures, while Venn diagrams were generated to illustrate shared and unique ASVs among groups. The Chao1, Shannon and Simpson (1-D) indices were used to visualize differences among groups in the alpha diversity analysis through the use of box plots. Beta diversity was evaluated by employing a PCoA plot that was based on the Bray–Curtis distance matrix to identify differences in community structure between samples. LEfSe analysis was conducted with an LDA score threshold of 4 or higher to identify biomarkers that were statistically significant. Furthermore, the classification performance and diagnostic value of the identified biomarkers were assessed through ROC curve analysis and the construction of a random forest model. Lastly, the FAPROTAX database (v1.2.1) was employed to predict the prospective ecological functions of the microbial community.

### Statistical analysis

Microbial data frequently deviate from a standard distribution. The Kruskal‒Wallis test was employed to compare the alpha diversity indices of multiple groups. Dunn's test was implemented for pairwise comparisons when significant differences were identified, with a significance threshold of *p* < 0.05. The Adonis test was employed to evaluate the differences in the overall community structure between the groups. To identify species with differences between groups (*p* < 0.05), the Kruskal‒Wallis test was employed by the LEfSe method, which subsequently employed LDA to estimate the effect size. The Mann‒Whitney U test was implemented to evaluate the functional prediction of FAPROTAX. A 95% confidence interval that did not cross 0 indicated *p* < 0.05.

## Results

### Characteristics of sequencing data

16S rRNA gene sequencing was implemented on all samples. After the identification of barcodes and the removal of low-quality sequences, a total of 4,249,151 full-length sequences of high quality were obtained. The average number of full-length sequences produced by each sample was 70,819, with a minimum of 49,505 sequences. A total of 2,820 ASVs were incorporated, comprising 26 phyla, 48 classes, 124 orders, 227 families, 531 genera and 825 species.

#### Overall composition of the microbial community

**Figure 1.**
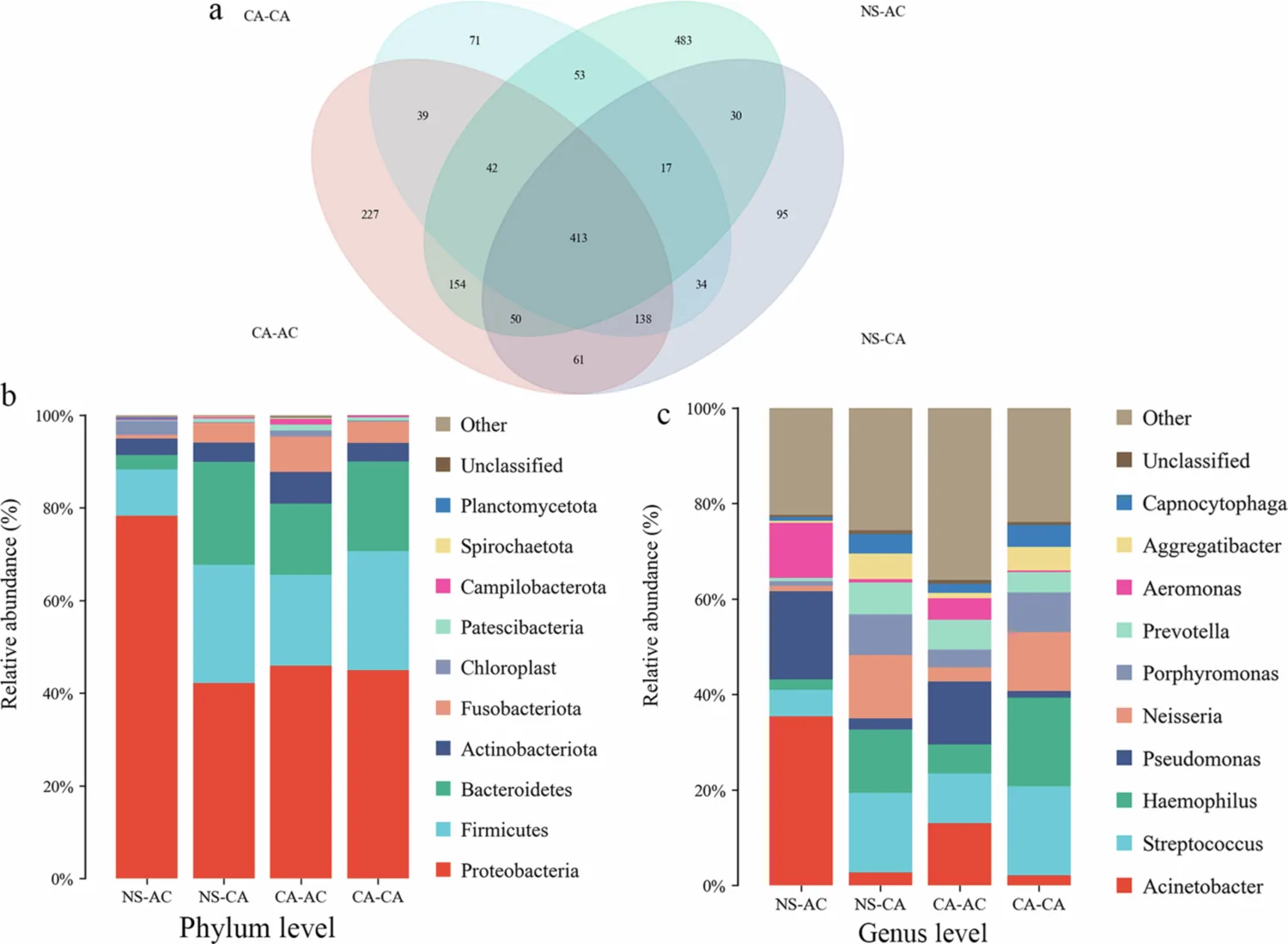
Analysis of the microbial community composition of the impression. (a) Venn diagram, (b) Relative abundance of bacterial communities at the phylum level, (c) Relative abundance of bacterial communities at the genus level. NS-AC: normal saline rinse + 2,000 mg/L available chlorine spray; NS-CA: normal saline rinse & chlorogenic acid (10 mg/mL mix + 60 mg/mL spray); CA-AC: 10 mg/mL chlorogenic acid rinse + 2,000 mg/L available chlorine spray; CA-CA: chlorogenic acid (10 mg/mL rinse & mix + 60 mg/mL spray).

A Venn diagram was generated to illustrate the shared and distinctive characteristics of the core microbial communities among various treatment groups (Figure 1a). The analysis demonstrated that the four groups shared 413 central ASVs. The respective groups of NS-AC, NS-CA, CA-AC and CA-CA contained 483, 95, 227 and 71 unique ASVs. It is important to note that the NS-CA and CA-AC groups shared the most ASVs, with a total of 662. These results indicate that the central microbial communities are influenced by a variety of treatment methods, which not only preserve the common substrate but also introduce unique members in their own unique manner.

Moreover, to further clarify the influence of various disinfection protocols on the microbial composition of the impression, we conducted an analysis of the relative abundances at the phylum and genus levels in all four groups. *Proteobacteria*, *Firmicutes* and *Bacteroidetes* were the three most prevalent phyla in all samples, collectively comprising more than 80% of the total community abundance. Intriguingly, the relative abundance of *Proteobacteria* was lower in the NS-CA and CA-CA groups than in the NS-AC and CA-AC groups. By contrast, *Firmicutes* and *Bacteroidetes* exhibited a higher abundance in the NS-CA and CA-CA groups (Figure 1b). The dominant microbial taxa across various treatment groups exhibited a distinct bipolar distribution at the genus level. The NS-AC and CA-AC groups exhibited a highly similar community composition, as did the NS-CA and CA-CA groups. The NS-AC group was distinguished by the predominance of *Acinetobacter*, *Pseudomonas* and *Aeromonas*, while the CA-AC group was characterized by *Acinetobacter*, *Streptococcus* and *Pseudomonas*. The NS-CA and CA-CA groups were characterized by *Streptococcus*, *Haemophilus* and *Neisseria* (Figure 1c). This pattern of distribution suggests that the dominant genera on the impression surface are primarily determined by the type of disinfectant used during the spraying process. AC and CA spraying not only altered the relative abundances of major phyla but also, more significantly, differentially shaped the dominant microbial community structures at the genus level.

### Analysis of microbial community diversity

**Figure 2.**
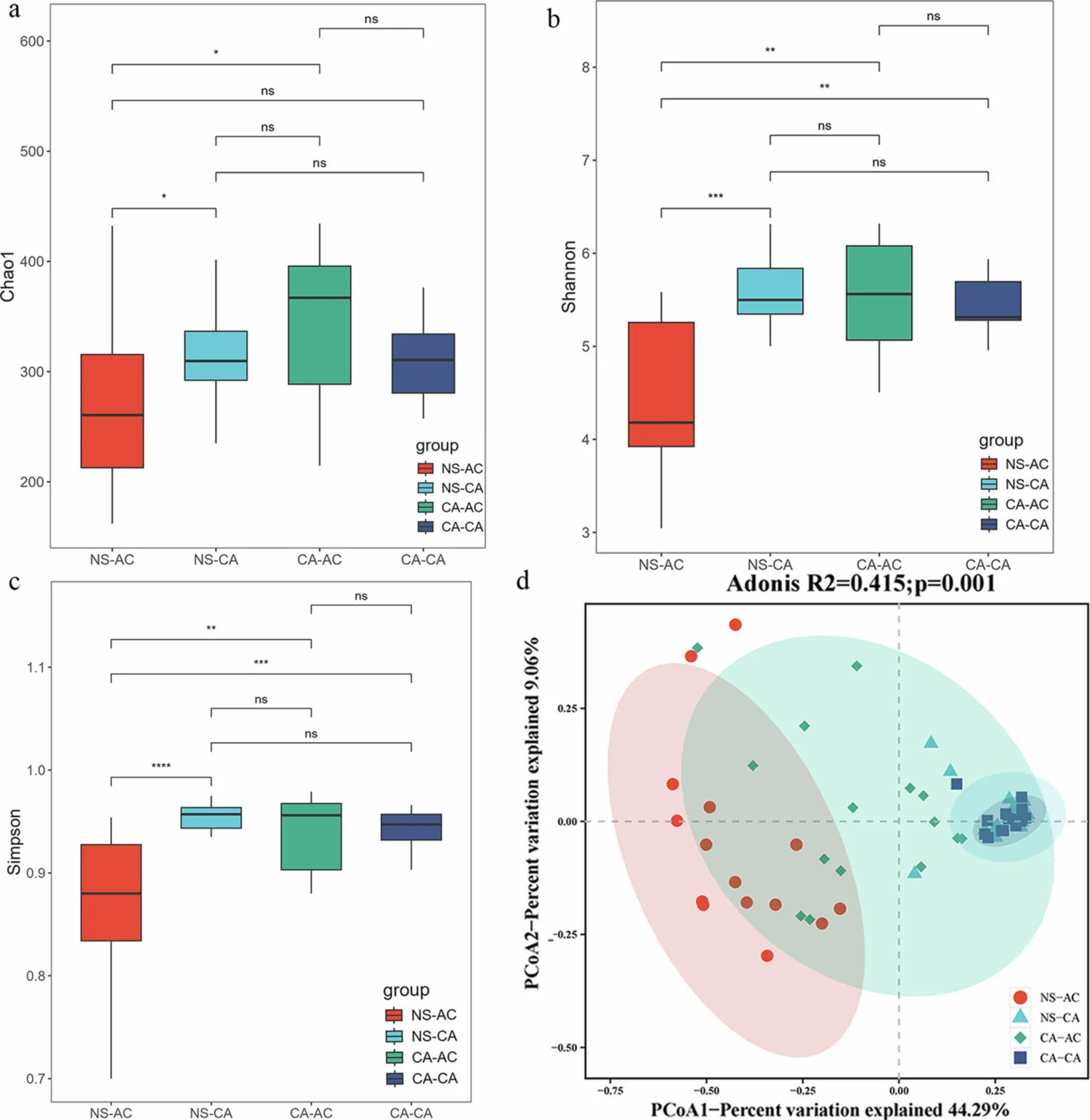
Diversity analysis of the microbial community of the alginate impression. Alpha diversity analysis: box plots of the Chao1 (a), Shannon (b) and Simpson (c) indices. Beta diversity analysis: (d) PCoA plot based on the Bray–Curtis distance. *p < 0.05, **p < 0.01, ***p < 0.001, ns: no significant difference. NS-AC: normal saline rinse + 2000 mg/L available chlorine spray; NS-CA: normal saline rinse & chlorogenic acid (10 mg/mL mix + 60 mg/mL spray); CA-AC: 10 mg/mL chlorogenic acid rinse + 2,000 mg/L available chlorine spray; CA-CA: chlorogenic acid (10 mg/mL rinse& mix + 60 mg/mL spray).

Compared to the NS-AC group, the Chao1 index was significantly higher in the NS-CA and CA-AC groups in the alpha diversity analysis (*p* < 0.05). Yet, no significant difference was observed between the CA-CA and NS-AC groups (Figure 2a). The Shannon and Simpson (1-D) indices exhibited more pronounced trends. Compared to the NS-AC group, both indices were significantly higher in the NS-CA, CA-AC and CA-CA groups (*p* < 0.01) (Figure 2b and c). Overall, these findings suggest that the NS-AC treatment contributed to a substantially reduced level of community diversity, particularly in terms of species evenness, when paired with the other disinfection protocols.

The first two principal components (PC1 and PC2) accounted for 53.35% of the total variation in community beta diversity, as shown by the Bray–Curtis distance-based PCoA (Figure 2d). In the positive region of the PC1 axis, samples from the NS-CA and CA-CA groups were closely concentrated, while samples from the NS-AC and CA-AC groups were predominantly distributed in the negative region, implying a distinct community separation. In the interim, the PERMANOVA verified that these inter-group differences were statistically significant (R^2^ = 0.415, *p* = 0.001). The microbial community structure on the impression surface was substantially altered by the various disinfection protocols, as evidenced by these results. The NS-CA and CA-CA groups exhibited a high degree of community similarity and stability, whereas the NS-AC and CA-AC groups exhibited greater variability. It is important to note this.

### Identification of key biomarkers

**Figure 3.**
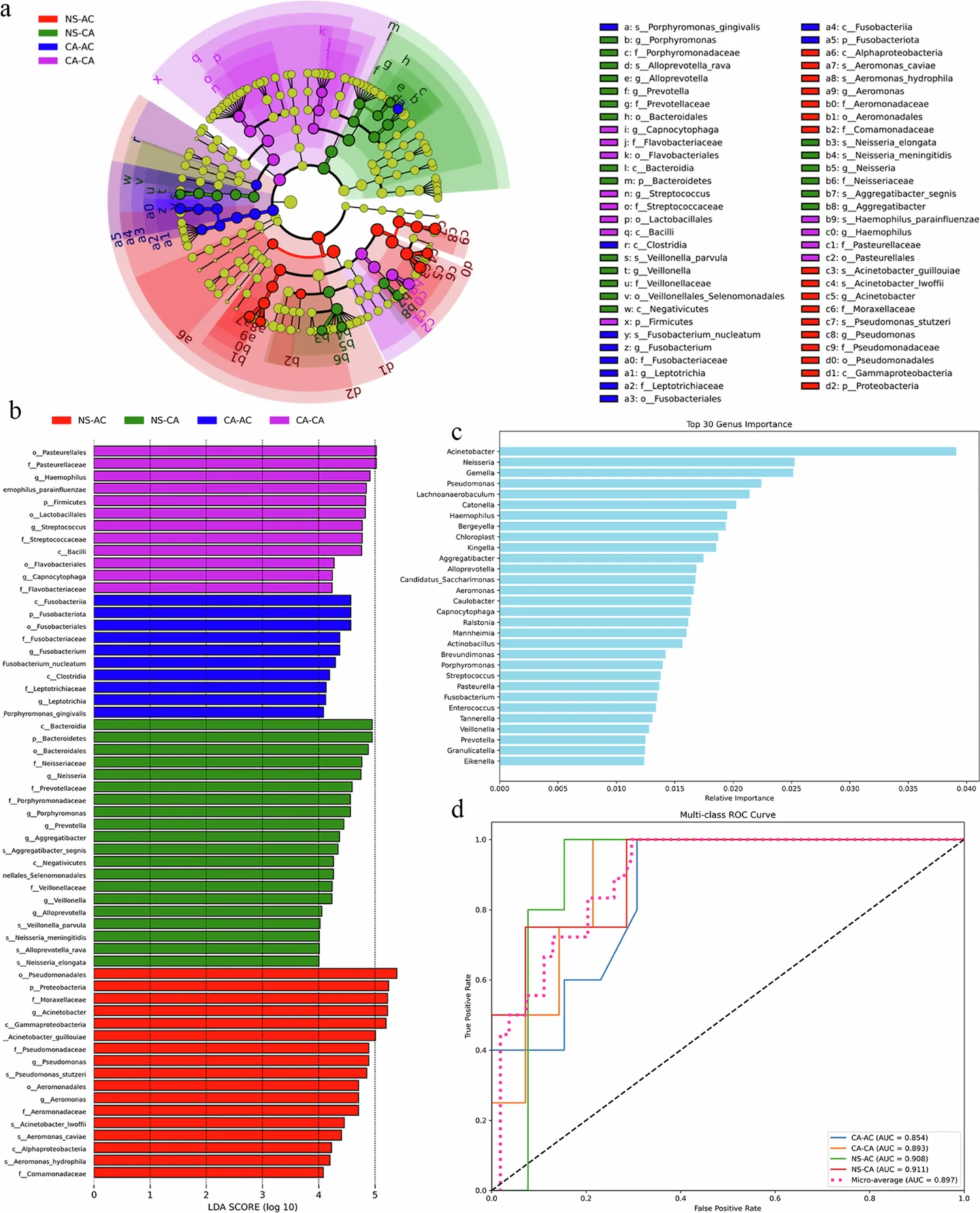
Key biomarkers identified by LEfSe analysis and the random forest model. (a) Cladogram generated by LEfSe showing taxonomic distribution of biomarkers; yellow nodes indicate microbes with no significant inter‑group differences at this level. (b) LDA score bar chart. (c) Random forest classification model. (d) ROC curve with AUC value. NS-AC: normal saline rinse + 2,000 mg/L available chlorine spray; NS-CA: normal saline rinse & chlorogenic acid (10 mg/mL mix + 60 mg/mL spray); CA-AC: 10 mg/mL chlorogenic acid rinse + 2,000 mg/L available chlorine spray; CA-CA: chlorogenic acid (10 mg/mL rinse& mix + 60 mg/mL spray). High-resolution versions of each subfigure are available in the Supplementary Materials (Supplementary Figures S3a–S3d).

In order to identify critical biomarkers associated with each disinfection treatment, LEfSe was implemented in accordance with the disparities in species abundance among groups. The disinfection treatments had a comprehensive impact on the microbial community structure, as evidenced by the cladogram, which showed that biomarkers for the four groups were widely distributed across multiple taxonomic levels, from phylum to genus (Figure 3a). The significantly enriched taxa in each group were further emphasized by the LDA score bar chart (LDA > 4, *p* < 0.05) (Figure 3b). The NS-AC group was distinguished by the enrichment of *Acinetobacter*, *Pseudomonas* and *Aeromonas* at the genus level. Alternatively, the NS-CA group exhibited a unique collection of biomarkers, such as *Neisseria*, *Porphyromonas*, *Prevotella*, *Aggregatibacter*, *Veillonella* and *Alloprevotella*. The CA-AC group was predominantly enriched for *Fusobacterium* and *Leptotrichia*, while the CA-CA group was dominated by *Haemophilus*, *Streptococcus* and *Capnocytophaga*. It is important to mention that the CA‑CA group was dominated by oral commensal microorganisms in comparison to the other groups. A random forest classification model was developed at the genus level to further assess the predictive capabilities of these biomarkers (Figure 3c). A total of 30 bacterial genera were identified as critical predictors that were capable of effectively discriminating among the four treatment groups. The AUC value was obtained through ROC curve analysis, which confirmed the high accuracy of these biomarkers in differentiating between the NS-AC, NS-CA, CA-AC and CA-CA groups (Figure 3d) (Supplementary Figure S3a-S3d).

### Prediction of microbial community ecological functions

**Figure 4.**
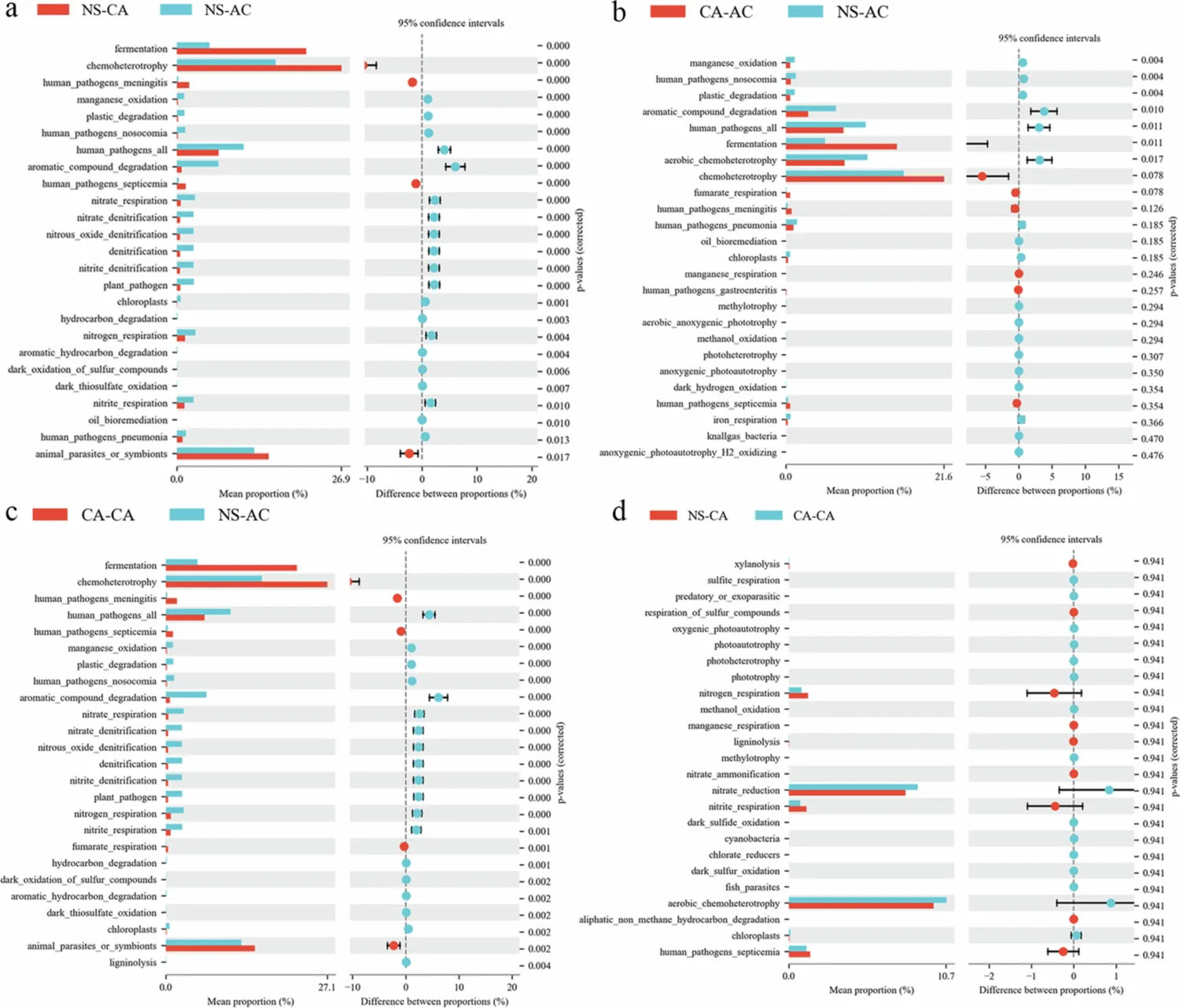
Analysis of inter-group differences in the predicted abundance of FAPROTAX functions. (a) NS-CA vs NS-AC; (b) CA-AC vs NS-AC; (c) CA-CA vs NS-AC; (d) NS-CA vs CA-CA. NS-AC: normal saline rinse + 2,000 mg/L available chlorine spray; NS-CA: normal saline rinse & chlorogenic acid (10 mg/mL mix + 60 mg/mL spray); CA-AC: 10 mg/mL chlorogenic acid rinse + 2,000 mg/L available chlorine spray; CA-CA: chlorogenic acid (10 mg/mL rinse& mix + 60 mg/mL spray). High-resolution versions of each subfigure are available in the Supplementary Materials (Supplementary Figure S4a-S4d).

Thus, to clarify the potential ecological roles of various disinfection treatments on the alginate impression microbial community, functional predictions were conducted using the FAPROTAX database (v1.2.1). The functional abundance differences between groups were compared using the Mann‒Whitney U test. The NS-AC group was chosen as the primary reference for comparisons with the other treatment groups in accordance with the research objectives (Figure 4a, b and c). In particular, the NS‑AC group exhibited significantly higher predicted abundances of *aromatic*_*compound_degradation* and *human_pathogens_all* compared to the other groups (*p* < 0.05). It is important to mention that the NS‑CA and CA‑CA groups showed significant ecological convergence and interchangeability, and the difference was not statistically significant (*p* > 0.05) (Figure 4d). As well, the CA-AC group demonstrated comparable functional variations and community structure distributions to the NS-AC group, and its functional spectrum was consistent across all four groups. In consequence, the functional prediction analysis did not include a comparison of the CA-AC group with other groups (Supplementary Figure S4a-S4d).

## Discussion

The oral microbiome is a significant component of the human microbiome and is a complex ecosystem that includes hundreds of microbial species [24,25]. Alginate impressions readily adsorb and retain a significant number of microorganisms, such as bacteria, viruses and fungi, when they penetrate the buccal cavity and come into contact with saliva, mucosa and tooth surfaces. Impressions are a potential vector for cross-infection in dental practice due to this characteristic [26,27]. At present, AC is the disinfectant that is employed most frequently in the preparation of alginate impressions. Nevertheless, research has indicated that AC's potential for long-term clinical use is restricted by its strong oxidative and corrosive properties, as well as its potential toxicity [28,29]. In this context, we discovered that the natural component CA can be employed as an alternative disinfectant. Our previous research has shown that CA is not only safe and environmentally friendly, but it also entirely inhibits the growth of four representative pathogenic bacteria [21,30]. Additionally, it does not damage alginate impressions. In the past, the primary endpoint for evaluating the efficacy of disinfection for alginate impressions was the CFU reduction rate, which was exclusively based on microbial culture methods. Nonetheless, this method is unable to account for the ecological influence of disinfectants on the entire microbial community that resides on the impression surface [31,32]. The potential risk of subsequent infection associated with impressions might be influenced by the microbial community structure that remains on the impression surface following disinfection. Therefore, the present study implemented 16S rRNA gene sequencing into impression disinfection research to resolve this methodological gap. The aim was to examine the impact of CA and AC disinfectants on the impression surface's overall microbial community. These results not only assessed the clinical safety of various disinfectants in terms of species composition, but also provided a scientific basis for the selection of impression disinfection methods that were of significant clinical relevance.

Initially, the microbial community structure of the NS‑AC group in this study exhibited typical dispersion characteristics, whereas the NS-CA and CA-CA groups exhibited more mature and stable community traits. Secondly, the NS-AC group was enriched with *Acinetobacter*, *Pseudomonas* and *Aeromonas* at the genus level. *Acinetobacter* and *Pseudomonas* are widely distributed in soil, water and hospital environments, while *Aeromonas* is predominantly found in freshwater environments [33–35]. These three genera are not typical oral commensals; however, they can be transmitted through tap water, operator contact, or contaminated dental instruments [36–39]. Their predominance on the impression surface indicates that the NS-AC treatment indiscriminately eliminated a significant portion of the oral commensal bacteria, thereby vacating ecological niches for these environmentally derived opportunistic pathogens. As a result, the disinfected impression may function as a potential infectious reservoir. *Acinetobacter* is a multidrug-resistant opportunistic pathogen that is environmentally derived and is recognized for its exceptional desiccation tolerance and adaptation to oxidative stress. It is also designated as a priority pathogen by the World Health Organization [40,41]. *Pseudomonas* is the second most prevalent Gram-negative bacterium associated with hospital-acquired infections, and it is a metabolically versatile environmental ‘survival expert’ that is distinguished by its robust biofilm-forming capacity and intrinsic drug resistance [42–44]. *Aeromonas*, a dominant genus in aquatic environments, has the ability to cause both intestinal and extraintestinal infections in immunocompromised individuals and possesses comprehensive stress response systems [35,45]. Collectively, these genera demonstrate robust metabolic capabilities, multidrug resistance and high environmental tolerance. Additionally, they are well-known opportunistic pathogens that are involved in nosocomial infections. Conversely, the NS‑CA and CA‑CA groups were characterized by oral commensals, including *Streptococcus*, *Haemophilus* and *Neisseria*, which are associated with significantly lower pathogenicity [46–49].

Additionally, the NS-AC group displayed a lower alpha diversity index than the other groups, and the beta diversity showed a discrete trend, suggesting a higher degree of inter-sample variability. Despite this, the NS-CA and CA-CA groups indicated a more concentrated beta diversity, which is a signal of a higher level of community stability and reproducibility. Genus-level biomarkers that effectively differentiated between treatment groups were identified through LEfSe analysis and random forest modeling. The AUC values of these biomarkers ranged from 0.8 to 0.9, suggesting a strong capacity to discern between various groups according to their biomarkers. Lastly, the NS-AC group achieved a synchronous and significant increase in the predicted abundances of functions related to *aromatic_compound_degradation* and *human_pathogens_all* at the functional level (*p* < 0.05). This functional profile is in close alignment with the ecological functions of the dominant genera that are enriched in this group. On the other hand, the NS-CA and CA-CA groups showed consistently lower abundances of these functions (*p* < 0.05). This implies that the functional profile is more balanced as a result of the simultaneous reduction of both *aromatic_compound_degradation* and *human_pathogens_all* in the community by CA treatment. More importantly, the NS-CA and CA-CA groups reported no statistically significant difference in the aforementioned functions (*p* > 0.05), demonstrating that these two disinfection protocols are functionally equivalent. The functional profile of the CA-AC group was intermediate, with characteristics that were more reminiscent of those of the NS-AC group.

The profoundly different antibacterial mechanisms of AC and CA treatments are the source of their distinct ecological effects. AC disinfection starts a rapid cell death by indiscriminately disrupting microbial cell walls, cell membranes and intracellular proteins and nucleic acids, thereby exerting a potent oxidizing effect [50–52]. While this non-selective oxidation is highly effective in its bactericidal action, it eliminates the majority of oxidant-sensitive microorganisms, thereby selectively enriching only a small number of taxa with high oxidative tolerance [53,54]. *Acinetobacter*, *Pseudomonas* and *Aeromonas* are precisely these types of bacteria; their robust antioxidant enzyme systems and adaptable oxygen metabolism facilitate their survival in the presence of severe oxidative stress [35,55–57]. Therefore, the fundamental principle of AC disinfection is the use of antioxidant capacity as the selection criterion, resulting in a microbial community that is dominated by opportunistic pathogens, has a dispersed structure and has reduced diversity. However, CA treatment employs a more subtle form of interference rather than wholesale destruction. Its antibacterial mechanisms are multi-targeted and consist of suppressing quorum sensing systems to prevent biofilm formation, disrupting membrane integrity and inhibiting key metabolic enzymes to interfere with energy metabolism and increasing bacterial membrane permeability, which results in the leakage of intracellular contents [58–61]. Ultimately, this multifaceted, milder approach selects for bacteria that are capable of maintaining metabolic activity and interspecies coexistence under CA exposure, thereby establishing a mature, stable and balanced microbial community.

The findings of this study necessitate a reassessment of the conventional criteria for evaluating the efficacy of disinfectants. Historically, AC has been widely recommended for impression disinfection due to its high sterilization rate [62,63]. Still, the AC treatment in this study was associated with a functional profile characterized by concurrent increases in the predicted abundances of *aromatic_compound_degradation* and *human_pathogens_all* on the impression surface, despite its high bactericidal efficacy. This means that the efficacy of impression disinfection should be assessed in addition to the disinfection rate on the microbial community structure. It is important to realize that the CA treatment not only effectively suppressed pathogen-related functions but also maintained a commensal-dominated community structure. This preliminary evidence supports the decision to expand the disinfection efficacy assessment from sterilization rate to community-level safety. In addition, the NS-CA and CA-CA groups obtained ecological outcomes that were nearly equivalent among the four treatment protocols that were evaluated. The NS-CA regimen provides the additional benefits of reduced material costs and simplified operational procedures. In this regard, it is advisable to prioritize the use of NS-CA treatment in future disinfection procedures and minimize the use of AC. Besides that, this study offers the first comprehensive evidence that CA disinfection is effective in killing bacteria, achieves stable and mature ecological regulation and does not affect the accuracy of alginate impressions, building upon our previous work [21]. The present study incorporates multiple evaluation dimensions to validate disinfectant performance, in contrast to the majority of previous investigations, which have only addressed isolated aspects of impression disinfection. Besides, the impression material may exhibit uniform distribution of CA that is incorporated during the mixing procedure. We hypothesize that this method may theoretically prevent contamination that is introduced during material storage and mixing. It may also assist in the reduction of disinfection dead zones that are caused by the intricate morphology of impressions. Even so, this concept is still a theoretical inference and calls for additional validation in future research.

In any case, it is imperative to recognize the numerous constraints of this investigation. Originally, all impression samples were obtained from orally healthy individuals, with the exception of patients who had common oral diseases, such as dental caries, periodontitis, or mucosal inflammation. Regardless of the fact that this design guaranteed that the ecological effects of various disinfection protocols were assessed under analogous baseline conditions, it is still uncertain whether the results apply to patient populations with a variety of oral pathologies. The applicability of CA-based disinfection in these clinical populations should be the focus of future research. Second, the experimental samples were collected uniformly 30 minutes following the application of the disinfectant. However, in the context of a hectic clinical practice, it may not always be possible to adhere strictly to this specific time frame. As such, in order to evaluate the temporal dynamics of the microbial community in the aftermath of disinfection, future research should include a variety of sampling time points. Third, the primary methodology employed in this investigation was 16S rRNA gene sequencing. The objective was to investigate the ecological implications of CA disinfection on the microbial community on alginate impression surfaces, rather than merely comparing total bacterial counts. Although previous studies have verified that CA effectively suppresses the growth of four representative strains on impressions using CFUs, the integration of qPCR into our current research could have provided additional supplementary data to evaluate the efficacy of disinfection. qPCR with genus-specific primers could be developed in the future to substantiate our primary findings. Also, the annotation coverage of the FAPROTAX database utilized in this study is restricted. As a result, in order to comprehensively elucidate the complete regulatory cascade of CA disinfection, which encompasses functional genes and metabolic outputs, a combination of multi-omics techniques, such as metagenomics, metatranscriptomics and metabolomics, will be required [64]. Ultimately, this was a single-center study in which all participants were recruited from the same geographic region. The oral microbiome structure may differ across populations from different regions, regardless of the fact that this design minimized confounding due to population heterogeneity. Given this, the disinfection efficacy and ecological effects of the NS-CA protocol should be validated through multi-center randomized controlled trials that employ sample sizes that are both larger and more geographically diverse.

## Conclusion

Our research demonstrates that CA disinfection results in a stable, functionally balanced and commensal-rich microbial community on alginate impressions, while AC disinfection generates a structurally simple, distinct community that is dominated by opportunistic pathogens. Among the groups, the NS-CA protocol exhibited cost‑effectiveness advantages and could be regarded as a viable alternative to traditional AC disinfection.

## Supplementary Material

Supplementary Material Figure S3d.tif

Supplementary Material Figure S4a.tif

Supplementary Material Figure S4c.tif

Supplementary Material Figure S4d.tif

Supplementary Material Figure S3a.tif

Supplementary Material Figure S4b.tif

Supplementary Material Figure S3b.tif

Supplementary Material Supplementary Figure.docx

Supplementary Material Figure S3c.tif

## Data Availability

The data that support the findings of this study are available from the corresponding author upon reasonable request.
