# Supplementary material for: The influence of chlorogenic acid disinfection on the oral microbiota of alginate impressions
Source: J Oral Microbiol. 2026 Sep 3;18(1):2727218. doi: 10.1080/20002297.2026.2727218 (PMC13543842; doi:10.1080/20002297.2026.2727218)
Supplement: Supplementary file 1 [file ZJOM_A_2727218_SM7175.docx]

Supplementary Figure S3a: Cladogram generated by LEfSe showing taxonomic distribution of biomarkers.

Supplementary Figure S3b: LDA score bar chart.

Supplementary Figure S3c: Random forest classification model.

Supplementary Figure S3d: ROC curve with AUC value.

Supplementary Figure S4a: Analysis of inter-group differences in the predicted abundance of FAPROTAX functions; (a) NS-CA vs NS-AC.

Supplementary Figure S4b: Analysis of inter-group differences in the predicted abundance of FAPROTAX functions; (b) CA-AC vs NS-AC.

Supplementary Figure S4c: Analysis of inter-group differences in the predicted abundance of FAPROTAX functions; (c) CA-CA vs NS-AC.

Supplementary Figure S4d: Analysis of inter-group differences in the predicted abundance of FAPROTAX functions; (d) NS-CA vs CA-CA.
